# Supplementary material for: The evolution of conditional moral assessment in indirect reciprocity
Source: Sci Rep. 2017 Feb 2;7:41870. doi: 10.1038/srep41870 (PMC5288800; doi:10.1038/srep41870)
Supplement: Supplementary Information [file srep41870-s1.pdf]

Supporting Information for

The evolution of conditional moral assessment in indirect reciprocity

Tatsuya Sasaki, Isamu Okada, Yutaka Nakai

This PDF file includes Text S1 and Table S1.

Text S1: Evolutionary dynamics for second-order social norms

We then focus on the 16 second-order social norms (except for one that prescribes unconditional defection given by BBBB (to unconditionally assign bad using Table 1). Compared to the case of the Staying norm, any of these 16 norms is less likely to invade a population of defectors. We note that since discriminators and defectors intend to refuse to help a bad recipient, $g_{Y,B} = g_{Z,B}$ holds. To analyze the replicator equations, we apply the expected payoffs, as in equation (5). For any of the 16 norms, equation (6) becomes

$$P_Z - P_Y = (1-e_1)[b(g_{Z,G} - g_{Y,G})z - c]g. \qquad \text{(S1)}$$

We consider small but non-zero errors, which yield $0 < g < 1$. When $g_{Z,G} - g_{Y,G} \le 0$, it follows that $P_Z - P_Y < 0$, and thus discriminators are dominated by defectors. When $g_{Z,G} - g_{Y,G} > 0$, this can result in, if any, a boundary equilibrium R with $z = z_R$, such that it satisfies

$$z_R = \frac{c}{(g_{Z,G} - g_{Y,G})b} \ge \frac{c}{b}. \qquad \text{(S2)}$$

equation (S2) implies that for all cases of the 16 second-order social norms, the threshold frequency for discriminators to successfully invade a population of defectors is at least the cost-to-benefit ratio c/b.

Scoring, Simple-standing, Stern-judging, and Shunning

We compare the effectiveness of Staying with the results of the most prevailing social norms. We specifically check the global dynamics of Scoring, Simple-standing, Stern-judging, and Shunning. First, we note that by definition the conditional probability that a donor is assessed as good when a potential recipient is good (that is, the first term in the sum in equation (3)) is the same as it is in equation (4) of Staying. The difference is in the second term in the sum.

For **Scoring**, we obtain

$$\begin{aligned} g_X &= \varepsilon g + \varepsilon(1-g) = \varepsilon, \\ g_Y &= e_2 g + e_2(1-g) = e_2, \\ g_Z &= \varepsilon g + e_2(1-g). \end{aligned} \tag{S3}$$

Since Scoring is a first-order social norm that depends only on what a donor did, the degrees of goodness in cooperators and defectors, g_X and g_Y, are independent of the recipient's degree of goodness g. A discriminator is assessed as good for a good recipient, with probability ε, or for a bad recipient, with the probability that he intentionally defects yet with assessment error e_2.

Next, for **Simple-standing**,

$$\begin{aligned} g_X &= \varepsilon g + (1-e_2)(1-g), \\ g_Y &= e_2 g + (1-e_2)(1-g), \\ g_Z &= \varepsilon g + (1-e_2)(1-g). \end{aligned} \tag{S4}$$

Simple-standing is the most tolerant norm, which is to assign a good image to a donor, irrespective of his/her actions to a bad recipient. Thus, the second term in the sum is the same as $(1-e_2)(1-g)$ over g_X, g_Y, and g_Z, in which case the donor is assessed as good only when no assessment error occurs.

Then, for **Stern-judging**,

$$g_X = \varepsilon g + (1-\varepsilon)(1-g),$$
$$g_Y = e_2 g + (1-e_2)(1-g), \quad \text{(S5)}$$
$$g_Z = \varepsilon g + (1-e_2)(1-g).$$

Stern-judging assigns a good image to those who refuse to help a bad recipient and a bad image to those who help a bad recipient. This leads to the second term in the sum for g_X. When a recipient is bad, unintentionally refusing help with no assessment error or intentionally giving help with assessment errors are both assessed as good. This conditional probability is $(1-e_1)e_2 + e_1(1-e_2) = 1-\varepsilon$.

Finally, for **Shunning**,

$$g_X = \varepsilon g + e_2(1-g),$$
$$g_Y = e_2 g + e_2(1-g), \quad \text{(S6)}$$
$$g_Z = \varepsilon g + e_2(1-g).$$

Shunning is the strictest case, in which a bad image is assigned to a donor irrespective of his/her actions toward a bad recipient. Thus, the second term in the sum is the same as $e_2(1-g)$ over g_X, g_Y, and g_Z, in which case the donor is assessed as good only when assessment errors occur.

Substituting equations (S3), (S4), (S5), or (S6) into equation (6), we obtain

$$P_Z - P_Y = (1-e_1)[(\varepsilon - e_2)bz - c]g. \quad \text{(S7)}$$

Considering $g > 0$, this results in a unique boundary equilibrium R with $z = z_R$, such that it is a repelling point and satisfies

$$z_R = \frac{c}{(\varepsilon - e_2)b} = \frac{c}{(1-e_1)(1-2e_2)b}. \quad \text{(S8)}$$

This leads to that when $(\varepsilon - e_2)b > c$, the unique equilibrium enters edge ZY ($x = 0$). Comparing equations (8) and (S8), it is obvious that the basin of attraction for node Z ($z = 1$) is wider under Staying than under the other four cases. Indeed, as the degrees of error, e_1 and e_2,

move toward 0, the fraction necessary for discriminators to emerge, z_R in equations (8) and (S8), converges to 0 and c/b, respectively. Thus, under Scoring, Simple-standing, Stern-judging, or Shunning, a sufficiently small cost-to-benefit ratio c/b is required for rare mutants of discriminators to successfully invade a population of defectors. In striking contrast to this, under Staying, rare mutants of discriminators can invade as long as assessment errors e_2 are very small.

Next, we turn to the payoff difference between cooperators and discriminators in equation (9). For Scoring, substituting equation (S3) yields

$$\begin{aligned} P_Z - P_X &= (1-e_1)[(e_2-\varepsilon)bz + c](1-g) \\ &= -(P_Z - P_Y)\frac{1-g}{g}. \end{aligned} \tag{S9}$$

Hence, there is a line consisting of fixed points with the same z-coordinate given by equation (S8). This line connects boundary fixed points Q on edge ZX ($y=0$) and R on edge ZY ($x=0$). We note that in contrast to R, Q is attracting along edge ZX. In particular, node Z is a saddle point, and node Y is a unique equilibrium that is asymptotically stable (see Fig. 2b). More details of these global dynamics can be explored by applying analogous arguments from refs. 5 and 39.

For Simple-standing or Shunning, substituting equation (S4) or (S6) yields the same results as in equation (10): $P_Z - P_X = (1-e_1)c(1-g) \geq 0$. For Stern-judging, substituting equation (S5) yields $P_Z - P_X = (1-e_1)[(\varepsilon - e_2)bz + c](1-g) \geq 0$. Thus, for the typical parameter settings, similarly to Staying, cooperators are dominated by discriminators and defectors, leading the population to converge to edge YZ ($x=0$). Thus, the global dynamics are qualitatively the same as those for Staying. However, the four social norms and the Staying norm differ quantitatively in the position of the repeller R, z_R (see Fig. 2c-e).

		Assessment: What does the donor image look like?								Action: What should donor do?			
Conditions	Recipient's image	G	G	G	G	B	B	B	B	G	G	B	B
	Donor's image	G	B	G	B	G	B	G	B	G	B	G	B
	Donor's action	C	C	D	D	C	C	D	D	–	–	–	–
Scoring		G	G	B	B	G	G	B	B	C	C	D	D
Shunning		Same as in Scoring				B	B	B	B	C	C	D	D
Leading eight	L1 (Standing)					G	G	G	B	C	C	D	C
	L2					B	G	G	B	C	C	D	C
	L3 (Simple-standing)					G	G	G	G	C	C	D	D
	L4					G	B	G	G	C	C	D	D
	L5					B	G	G	G	C	C	D	D
	L6 (Stern-judging)					B	B	G	G	C	C	D	D
	L7 (Strict-standing)					G	B	G	B	C	C	D	D
	L8					B	B	G	B	C	C	D	D
Staying						**P**	**P**	**P**	**P**	C	C	D	D

Table S1. Social norms: Staying, Scoring, Shunning, and the leading eight. "G" and "B" describe good image and bad image, respectively. "C" and "D" denote an action to help and to refuse to help, respectively. "P" means that the donor's image remains unchanged. With Scoring[19,20,39-41], whether to help or not determines the donor's image. When a potential recipient has a good image, the leading eight strategies[23,24] all have the same assessment as Scoring. Coding of the leading eight, L1 to L8, is the same as assigned in ref. 43. L1 is the original "Standing"[1,21,22], which is viewed as a third-order social norm. Only rules L1 and L2 have different action rules, which prescribe cooperation when both donor and recipient have bad

images. L1 (Standing) and L3 (Simple-standing) differ in that part of the action rule and also in the assessment of donors who refuse to help a bad recipient. Shunning[5,42] and Scoring do not belong to the leading eight, as Shunning could not achieve a sufficiently high degree of cooperation at the equilibrium state[38].
